# Daidzein and Equol: Ex Vivo and In Silico Approaches Targeting COX-2, iNOS, and the Canonical Inflammasome Signaling Pathway

**DOI:** 10.3390/ph17050647

**Published:** 2024-05-16

**Authors:** Yazmín K. Márquez-Flores, Elizdath Martínez-Galero, José Correa-Basurto, Yudibeth Sixto-López, Isabel Villegas, María Á. Rosillo, Ana Cárdeno, Catalina Alarcón-de-la-Lastra

**Affiliations:** 1 Departamento de Farmacia, Escuela Nacional de Ciencias Biológicas, Campus Zacatenco, Instituto Politécnico Nacional, Av. Wilfrido Massieu s/n Col. Zacatenco, Mexico City C.P. 07738, Mexico; emartinezga@ipn.mx; 2 Laboratorio de Diseño y Desarrollo de Nuevos Fármacos y Productos Biotecnológicos, Escuela Superior de Medicina, Instituto Politécnico Nacional, Plan de San Luis y Díaz Mirón s/n, Col. Santo Tomas, Mexico City C.P. 11340, Mexico; jcorreab@ipn.mx (J.C.-B.); ysixtol@ipn.mx (Y.S.-L.); 3 Departamento de Química Farmacéutica y Orgánica, Facultad de Farmacia, Campus de Cartuja, Universidad de Granada, 18071 Granada, Spain; 4 Department of Pharmacology, Faculty of Pharmacy, University of Seville, Professor García González Street 2, 41012 Seville, Spain; villegas@us.es (I.V.); anacardeno@us.es (A.C.); calarcon@us.es (C.A.-d.-l.-L.)

**Keywords:** soy, daidzein, equol, docking, inflammasome, inflammation

## Abstract

Background: The inflammasome is a cytosolic multiprotein complex associated with multiple autoimmune diseases. Phytochemical compounds in soy (*Glycine max*) foods, such as isoflavones, have been reported for their anti-inflammatory properties. Aim: the anti-inflammatory activity of DZ (daidzein) and EQ (equol) were investigated in an ex vivo model of LPS-stimulated murine peritoneal macrophages and by molecular docking correlation. Methods: Cells were pre-treated with DZ (25, 50, and 100 µM) or EQ (5, 10, and 25 µM), followed by LPS stimulation. The levels of PGE_2_, NO, TNF-α, IL-6, and IL-1β were analyzed by ELISA, whereas the expressions of COX-2, iNOS, NLRP3, ASC, caspase 1, and IL-18 were measured by Western blotting. Also, the potential for transcriptional modulation by targeting NF-κB, COX-2, iNOS, NLRP3, ASC, and caspase 1 was investigated by molecular docking. Results: The anti-inflammatory responses observed may be due to the modulation of NF-κB due to the binding of DZ or EQ, which is translated into decreased TNF-α, COX-2, iNOS, NLRP3, and ASC levels. Conclusion: This study establishes that DZ and EQ inhibit LPS-induced inflammatory responses in peritoneal murine macrophages via down-regulation of NO and PGE_2_ generation, as well as the inhibition of the canonical inflammasome pathway, regulating NLRP3, and consequently decreasing IL-1β and IL-18 activation.

## 1. Introduction

The inflammasome is a cytosolic multiprotein complex with intense inflammatory activity. Thus, it is highly regulated at the transcriptional and post-transcriptional levels [1].

**Figure 1:**
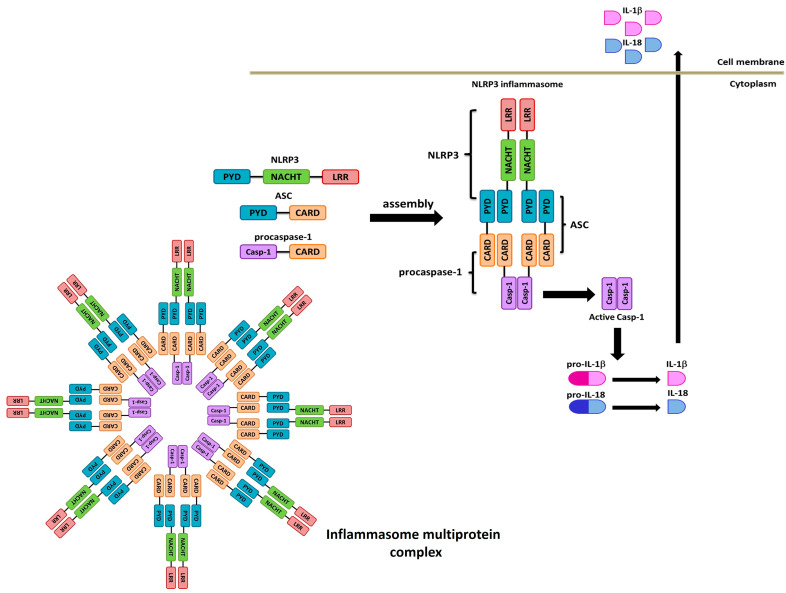
NLRP3 inflammasome complex.

Dysregulated inflammasome activity is associated with intermittent inflammatory and autoimmune responses, which leads to multiple autoimmune diseases and complex syndromes, including gout, type 2 diabetes, rheumatoid arthritis (RA), and inflammatory bowel disease (IBD); therefore, their regulation permits maintaining immune system homeostasis since, for example, an important consequence of the activation of NLRP3 (Figure 1) increases the levels of the inflammatory cytokines IL-1β and IL-18 [2,3,4].

The inflammasome commonly contains some essential components: a sensor protein, the adapter protein apoptosis-associated speck-like protein containing an activation and recruitment domain (CARD) domain (ASC), the inflammatory protease caspase 1, and a nucleotide-binding-domain (NOD)-like receptor (NLR) member, for example, NLRP3 (Figure 1) [5,6]. The activated sensor recruits ASC through homophilic interactions of pyrin domains, and ASC associates with procaspase 1 via CARD-CARD interactions, a step needed to induce the caspase 1 activation [3]. Once activated, it cleaves the biologically inactive interleukin (IL)-1β and IL-18 precursors, generating their mature forms, which are eventually secreted [6,7].

Both NLRs and the TLRs (Toll-like receptors) can mediate the eukaryotic cells’ recognition of PAMPs (pathogen-associated molecular patterns), such as lipopolysaccharide (LPS), leading to the activation of host cell signaling pathways and subsequent innate and adaptive immune responses [8]. Specifically, in macrophages, the TLRs activated by the LPS [9,10] induce multiple pathways involved in the production of pro-inflammatory chemokines and cytokines, such as TNF-α, IL-1β, IL-6, IL-17, IL-18, among others, as well as regulatory enzymes such as cyclooxygenase (COX)-2 and inducible nitric oxide synthase (iNOS), which are responsible for the overproduction of prostaglandin E2 (PGE_2_) and nitric oxide (NO), respectively [11,12,13,14]. The regulation of inflammatory gene expression plays a pivotal role in maintaining immune homeostasis; the transcription of pro-inflammatory genes is triggered by various inflammatory stimuli [15]. The NF-κB, a family of inducible transcription factors, mediates the inflammatory response by inducing the expression of several pro-inflammatory genes, such as cytokines (IL-1, IL-2, IL-6, IL-8, IL-12, TNF-α), chemokines (IL-18, MCP-1, etc.), some inflammasome components (NLRP3, Pro-IL-1β) [16], and even NF-κB is implicated in the transcription of inflammatory enzymes, such as iNOS and Ciclooxygenase-2 [16,17]. Some natural products have been reported as NF-κB inhibitors and, consequently, with anti-inflammatory activity by decreasing the expression of proinflammatory components [18,19,20].

Daidzein (DZ) (7-Hydroxy-3-(4-hydroxyphenyl) chromen-4-one) (Table 1) is an isoflavone of soy (*Glycine max*) foods [21]. Chemically, soy isoflavones are commonly β-glycosides that are not absorbed along the gastrointestinal tract and require hydrolysis to increase their bioavailability and subsequent metabolism. Hydrolysis occurs along the entire length of the intestinal tract by the action of the brush border membrane and bacterial β-glucosidases. Then, aglycones are released and metabolized. The formation of equol (EQ) (7-hydroxy-3-(4′-hydroxyphenyl)-chroman) (Table 1) from DZ occurs via a pathway that involves the construction of the intermediate dihydrodaidzein [22,23]. EQ has a unique structure characterized by a chiral carbon atom at C-3 of the furan ring, existing in two distinct enantiomeric forms: R- (R-EQ) and S-equol (S-EQ). Magee (2011) reports that human intestinal bacteria exclusively synthesize the S-EQ enantiomer from DZ, and only approximately 30–40% of the adult population could perform this transformation [24].

Abnormal levels of inflammation may lead to chronic inflammatory conditions, such as rheumatoid arthritis (RA), IBD, psoriasis, diabetes mellitus, chronic kidney disease, cancer, non-alcoholic fatty liver disease, and autoimmune and neurodegenerative disorders. Therefore, diverse research groups continuously search for molecules with anti-inflammatory responses [25,26,27].

This exploration includes phytochemical compounds in soy (*Glycine max*) foods, such as isoflavones. Hence, this current research investigated the anti-inflammatory activity of DZ and EQ, soy isoflavones, in an ex vivo model of murine peritoneal macrophages stimulated with LPS.

In vitro and in vivo studies have revealed that DZ is a highly active antioxidant molecule with anti-estrogenic, anti-cancer, anti-inflammatory, anti-diabetic, and cardioprotective effects [28,29]; however, recent reports report that EQ has superior bioactivity than that of other isoflavones, including DZ [30,31,32].

Considering this background, this present study explored the molecular mechanisms of DZ and EQ measuring macromolecules related to canonical inflammasome signaling pathways and other critical inflammatory mediators using an LPS-stimulated inflammatory response in murine peritoneal macrophages. Also, docking studies of DZ or EQ on COX-2, iNOS, NLRP3, ASC, and caspase 1 were carried out to decipher the target modulation that explains the biological results.

## 2. Results

### 2.1. Cell Viability

**Figure 2:**
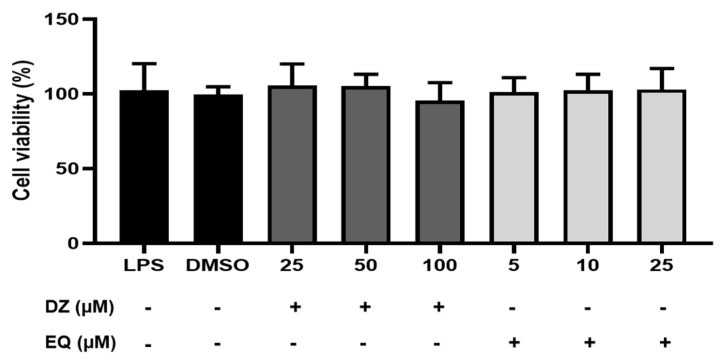
Cell viability percentage after DZ or EQ exposition. Values are represented as means ± SEM for at least three independent experiments. *p* = 0.3299. No statistical significances were observed.

The effects of DZ and EQ on cell viability were obtained by sulforhodamine B assay (Section 4.2.3) after 18 h of incubation. As shown in Figure 2, the cells treated with DZ (25, 50, 100 μM), S-EQ (5, 10, 25 μM), or LPS were not significantly reduced (*p* = 0.3299), indicating that compounds do not influence cell viability at the concentrations used in the study.

### 2.2. DZ and EQ Down-Regulated COX-2 Expression and PGE_2_ Levels

**Figure 3:**
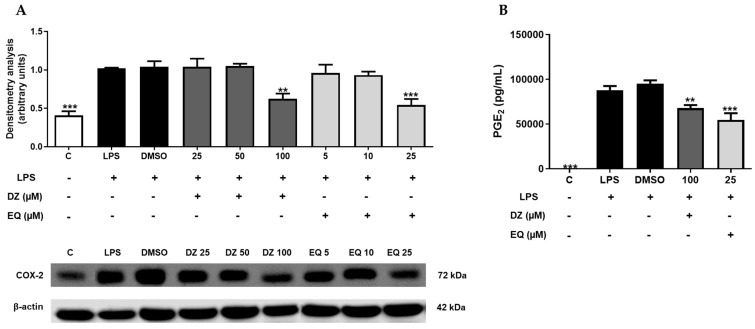
DZ and EQ effect on critical inflammatory mediators using an LPS-stimulated inflammatory response in murine peritoneal macrophages. (**A**) Densitometry analysis of COX-2 protein expression. C represents untreated control cells. (**B**) PGE_2_ levels in supernatants collected from LPS-stimulated macrophages. Data shown are means ± SEM for three independent experiments as a minimum. ** *p* < 0.01; *** *p* < 0.001, which are significantly different from LPS control cells.

In cells treated with DZ 100 µM (** *p* < 0.01) and S-EQ 25 µM (*** *p* < 0.001) (Figure 3A), a significant down-regulation of COX-2 expression was observed. In the same way, both treatments were able to down-regulate PGE_2_ levels, with the EQ treatment being the most potent one (** *p* < 0.01 and *** *p* < 0.001, DZ 100 µM, and EQ 25 µM, respectively) (Figure 3B).

### 2.3. Effects of DZ and EQ on Nitrite Production and iNOS Overexpression

**Figure 4:**
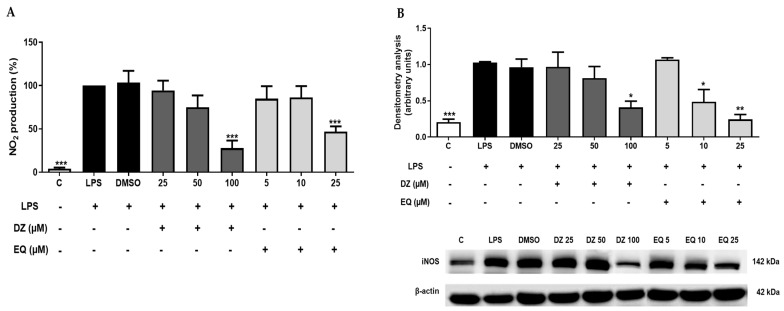
DZ and EQ effect on NO and iNOS enzyme levels in murine peritoneal macrophages. (**A**) Nitrite concentration was measured using the Griess method. (**B**) Densitometry analysis of iNOS protein expression. C represents untreated control cells. Data are represented as means ± SEM for 3 independent experiments as a minimum. * *p* < 0.05; ** *p* < 0.01; *** *p* < 0.001 versus LPS control cells.

It is well known that macrophages express TLR on their cell surface, which binds to LPS and can induce NO release [33]. First, the nitrite concentration was normalized, considering the LPS group to be 100% (Figure 4A). As observed, LPS stimulation increased the production of nitrites compared to that of untreated cells (*** *p* < 0.001), an effect also related to an increment (*** *p* < 0.001) of iNOS protein expression (Figure 4B). In both cases, DMSO induced an LPS-similar behavior.

With regard to DZ (100 µM) and EQ (25 µM), both treatments reduced nitrite production by 27.85 and 46.87%, respectively (*** *p* < 0.001), and decreased iNOS protein expression compared with the that of the LPS group. EQ at 10 µM (* *p* < 0.05) also down-regulated iNOS protein levels after LPS stimulation in murine peritoneal macrophages (Figure 4B).

### 2.4. DZ and EQ Down-Regulated Inflammatory Mediators Induced by LPS

**Figure 5:**
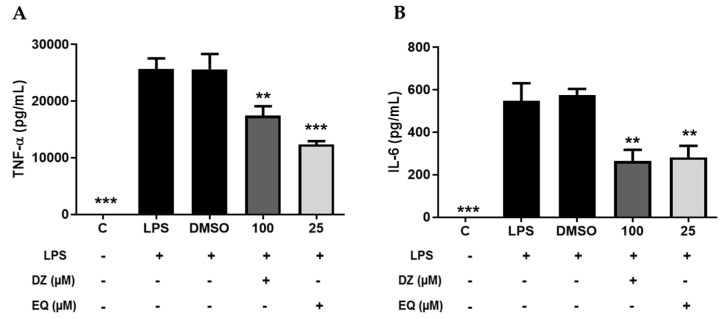
Down-regulation of TNF-α and IL-6 by DZ and EQ treatments. ELISA TNF-α (**A**) and IL-6 (**B**) quantification (pg/mL). Values are represented as means ± SEM for at least three independent experiments. ** *p* < 0.01; *** *p* < 0.001 versus LPS control.

TNF-α and IL-6 are pro-inflammatory cytokines involved in many inflammatory diseases. The TNF-α and IL-6 levels in supernatants collected from macrophages treated with DZ or EQ show a decrease in the levels of TNF-α and IL-6 (Figure 5A,B).

### 2.5. DZ and EQ Inhibit the Canonical Inflammasome Signaling Pathway

**Figure 6:**
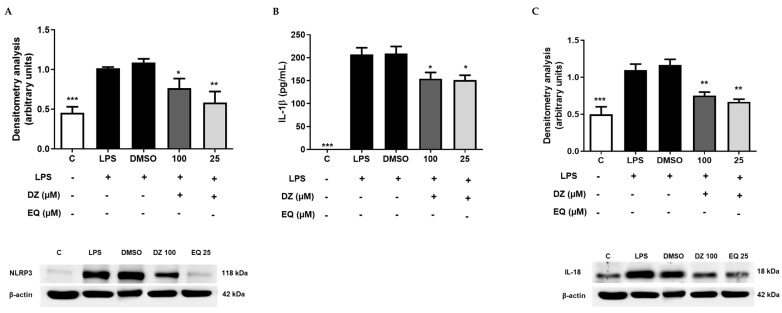
DZ and EQ effect on NLRP3 canonical signaling pathway in murine peritoneal macrophages. (**A**) Densitometry analysis of NLRP3 protein expression. C represents untreated control cells. (**B**) IL-1β secretion was determined by the ELISA method. (**C**) IL-18 densitometry analysis. C represents untreated control cells. Data are reported as means ± SEM for three independent experiments as a minimum. * *p* < 0.05, ** *p* < 0.01 and *** *p* < 0.001 significantly different from LPS treated control cells.

The most characterized NLR member of the inflammasome signaling pathway is the NLRP3. Considering previous ex vivo results, we evaluate the effect of the most potent concentrations of DZ (100 µM) and EQ (25 µM) in the NLRP3/ASC adaptor/Caspase 1 inflammasome signaling pathway. The results showed that DZ (* *p* < 0.05) and EQ (** *p* < 0.01) were able to down-regulated NLRP3 protein expression (Figure 6A). Nevertheless, there were effects on procaspase 1 and caspase 1 expression (ASC), suggesting that DZ and EQ may not favor caspase activation despite ASC’s importance regarding inflammasome activation [34].

This suggests that the decreasing IL-1β and IL-18 levels (Figure 6B,C) probably result from NLRP3 down-regulation.

### 2.6. Docking Studies of DZ and S-EQ on COX-2

Table 1 shows the values of free energies (∆G) (Kcal/mol), the constant inhibition (Ki), and the interactions between residues and DZ or EQ. Also, to determine the possible relationship between the affinity and the ligands’ structural features, we analyzed the primary complex’s interactions using AutoDock Tools 1.5.6 [35] and the Discovery Studio v17.2 Software (Dassault Systèmes BIOVIA, Waltham, MA, USA, 2016).

**Figure 7:**
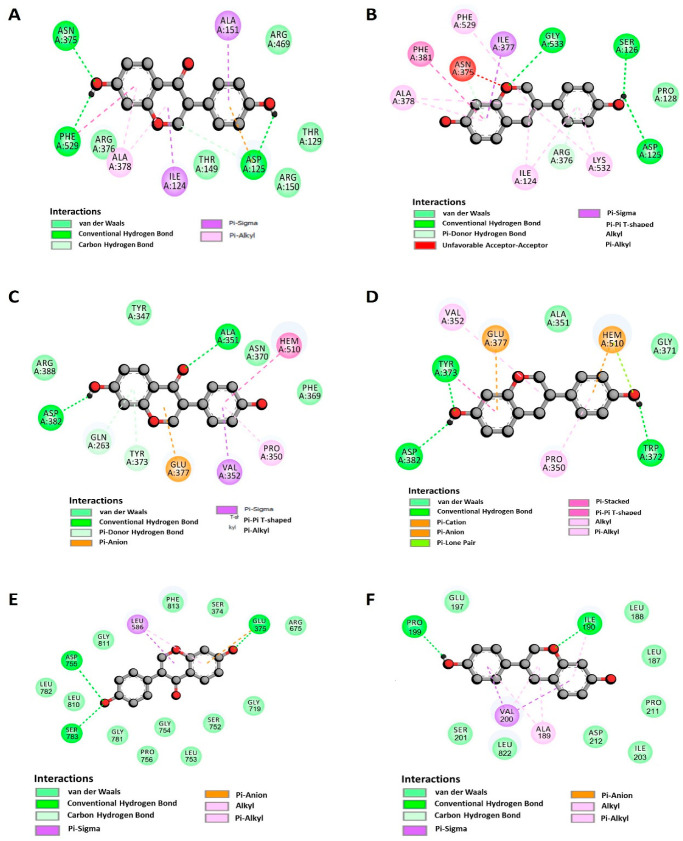
DZ and S-EQ docking interactions. COX-2 interactions with (**A**) DZ and (**B**) S-EQ. iNOS interactions with (**C**) DZ and (**D**) S-EQ. NLRP3 interactions with (**E**) DZ and (**F**) S-EQ. The circles represent the closest interactions. The images were obtained by Discovery Studio v17.2.

Both compounds showed high affinity on the COX-2 protein, the complex’s association to DZ, and S-EQ (∆G = −8.61 and −8.57 kcal/mol, respectively). According to our results, DZ forms a hydrogen bond with Asp125, Arg150, Asn375, and Phe529 (Figure 7A). Additionally, DZ interacts with Ala378. S-EQ shows a different profile of interacting residues such as Ser126, Pro128, Ile377, Lys532, and Gly533; however, DZ and S-EQ share interactions with Ile124, Asp125, Asn375, Arg376, Ala378, Phe381, and Phe529 (Figure 7A,B).

### 2.7. Docking Studies of DZ and EQ on iNOS

Related to iNOS complexes, DZ reaches Asp382 through its benzene ring connected by a pyran ring (Figure 4C). In this sense, the affinity for DZ and S-EQ was ∆G = −7.56 and −8.60 kcal/mol, respectively. Both compounds targeted the enzyme’s HEME group (Figure 7C,D).

### 2.8. Docking Results for NLRP3

As is seen in Table 1, all the compounds showed similar affinity to this protein, with ∆G values of −7.80 and −7.50 (DZ and S-EQ, respectively). DZ was bound to residues of the NACH domain (Ser374 and Glu375) and residues belonging to LRR domain 1 (Ser752, Leu753, and Gly754), 3 (Leu810 and Gly811), and 7 (Gly915, Leu916, and Ser917) (Figure 7E). S-EQ bounded to residues Pro199, Val200, Ser201, and Leu822 (Figure 7F).

### 2.9. Docking Results for ASC and Caspase 1

Docking results showed that S-EQ was the most favorable bound on caspase 1 (∆G = −5.95 kcal/mol). However, it is essential to note that only DZ (∆G = −5.57 kcal/mol) binds residues closely to ASC PYD: Asp10, 48, 51, and 54, as well as Glu13 (acidic surface patch); and residues Lys21 and Arg38 and 41 (basic patch) [36]. In another way, S-EQ was bound to the CARD domain. This domain binds to caspase 1 rather than interacting with the identical residues, except for the reason that the first interacts with Lys161 and the last interacts with Phe163.

Otherwise, compounds were docked on pro-caspase 1 and caspase 1, with DZ being the compound that was more affine, with ∆G values of −7.23 kcal/mol and −7.60 kcal/mol, respectively.

### 2.10. Docking Results for NF-κB

**Figure 8:**
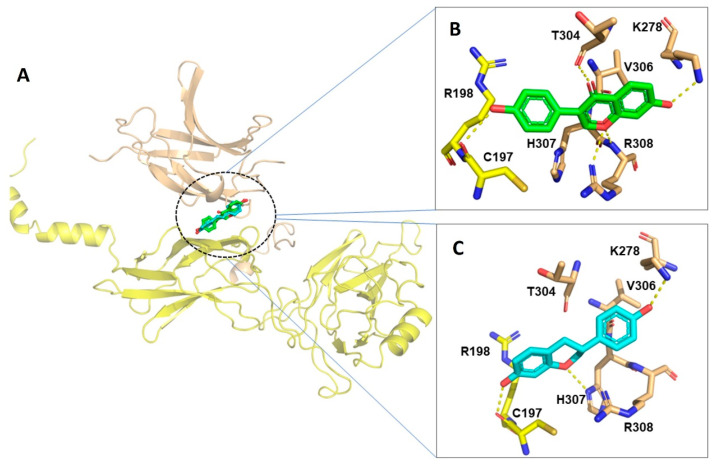
Molecular docking of DZ and S-EQ on NF-κB P50-P65 dimer (**A**). DZ (**B**) and EQ (**C**) binding mode and molecular interactions were established with NF-κB P50-P65 dimer.

Both DZ and EQ were able to reach the interface between P65 and P50 of NF-κB (Figure 8A), interacting with the residues of both monomers. No significant differences in the binding free energy were observed; DZ and EQ were bound with an energy of around −5.4 kcal/mol. However, there were marked differences in the binding mode. DZ interacted with residues of the P65 NF-κB by the oxygen of the phenyl group, forming a hydrogen bond with Cys197. At the same time, the chroman ring began numerous hydrogen bonds with residues of the P50 NF-κB, i.e., the oxygen of the carbonyl group formed a hydrogen bond with Thr304 and the oxygen of the ether group with Arg308. Finally, the oxygen of the hydroxyl group formed a hydrogen bond with Lys278 (Figure 8B). Conversely, EQ interacted with P65 NF-κB residues through the chroman moiety, and the hydroxyl of the chroman ring formed a hydrogen bond with Cys197 and interacted by non-bonding interactions with Arg198. In contrast, the oxygen ether of the chroman group formed a hydrogen bond with His307 of the P50-NF-κB monomer, and the oxygen of the phenyl group stabilized the whole structure by a hydrogen bond with Lys278 (Figure 8C).

### 2.11. Log p Value Estimation and Electrostatic Potential Map of DZ and EQ

The values obtained by the ALOGPS 2.1 program showed that DZ (log *p* = 2.77) slightly increases its lipophilicity when converted to EQ (log *p* = 2.91). Dihydrodaidzein and Cis/trans-isoflavan-4-ol, intermediaries of DZ metabolism, showed log *p* values of 2.77 and 1.82, respectively.

**Figure 9:**
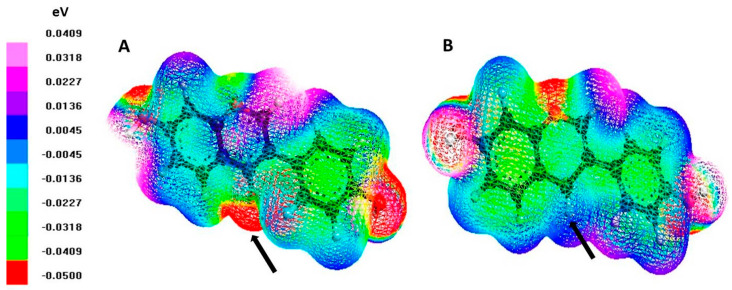
Electrostatic potential map. The color scale shows the electrostatic potential map of (**A**) DZ and (**B**) EQ. The white color is related to the most positive electronegativity potential, while the red color indicates the most negative one. The arrow indicates position 4 of the flavone.

Regarding the electrostatic potential of DZ and EQ, we obtained the map of the electron density surface, in which the red color represents the regions with the highest electronegativity, decreasing towards the white color according to less electronegativity (Figure 9).

As shown, converting DZ to S-EQ decreases the electronegativity of flavone, which is composed of two benzene rings connected by a heterocyclic pyran ring. This effect is more evident in the ketone in position 4 (arrow marked), a functional group lost during the DZ metabolism.

## 3. Discussion

Since the 1980s, molecular docking has been the most common computational structure-based drug design technique. Because discovering new drugs is a complex process, this permits the understanding and prediction of molecular recognition structurally and energetically. Its accuracy is about 1.5–2 Å with a 70–80% success range. However, it is important to consider the limitations of accurate binding energies and other parameters such as the solvent and the flexibility of the macromolecule used [37]. We used the docking analysis to correlate the ligand interactions and the ex vivo data in a murine peritoneal macrophage model.

Moreover, abnormal macrophage activation, particularly in its polarization towards the M2 phenotype, is implicated in the pathogenesis of immuno-inflammatory diseases such as systemic lupus erythematosus and rheumatoid arthritis, promoting the synovitis, cartilage, and bone destruction characteristic of these diseases [38,39].

Macrophages that are LPS-activated are associated with an imbalance in the cytokine network, stimulating the secretion of proinflammatory Th1 and Th17 cytokines, such as IL-6, IL-1β, and TNF-α, among others [40,41,42].

They also induce overexpression of the COX-2 enzyme responsible for the overproduction of PGE_2_, which participates in the inflammatory process through EP4 receptor activation [43]. In addition, after LPS stimulation, iNOS expression is induced, and high levels of NO are produced, acting as an intracellular messenger that modulates ROS production [44]. In agreement with these observations, our data showed a marked increase in these pro-inflammatory markers.

Our results show that DZ and EQ presented significant effects regarding proteins (COX-2, iNOS, NLRP3) involved in inflammatory activities. Also, DZ and EQ prevented the progression of the cellular damage induced by LPS in murine peritoneal macrophages, exhibiting S-EQ results that were better than those of DZ Gut bacteria chemically reduce DZ during digestion to convert it into the highly estrogenic S-EQ, non-estrogenic O-desmethylangolensin, or dihydrodaidzein. This chemical conversion is essential for favored absorption, better bioavailability, and estrogenic activity [45].

In this work, the peritoneal macrophages treated with LPS showed increased PGE_2_ levels and up-regulated COX-2 expression, the most crucial source of prostanoid formation during inflammation and cancer [46,47].

Two hypotheses were explored using a molecular docking approach to rationalize the experimental findings. First, it was hypothesized that both DZ and EQ could be directly bound to the studied proteins. Second, it was hypothesized that DZ and EQ could modulate the activity of NF-κB, a transcriptional factor that can positively regulate the transcription of the studied proteins [17,32]. Hence, the binding of EQ and DZ was explored by molecular docking on NF-κB, as mentioned below.

In this sense, according to docking studies, none of the compounds interacted with the HEME group in the COX-2 active site, opening the possibility that DZ and EQ bind to other hydrophobic sites in the enzyme nearest to the catalytic site, like methoxytetrahydropyran derivates and benzoxalzoline phytochemical derivate compounds, which act as allosteric modulators [48]. However, both compounds’ ability to down-regulate COX-2 protein expression was evident due to their affinity properties on the COX-2’s transcriptional factor and the consequent PGE_2_ levels, one of the principal bioactive PGs. These results are consistent with those from other studies, where DZ and EQ inhibited the expression of COX-2 protein and PGE_2_ levels in LPS RAW264.7, J774, and BV2-activated cells [49,50,51].

Regarding NO production, there are in vivo results in which EQ administration attenuates NO levels and iNOS gene expression in peritoneal adherent cells isolated from LPS-treated mice, suggesting that EQ could block Akt pathways with a subsequent down-regulation of NF-κB activity [52]. Also, Blay et al. tested the effect of some isoflavones from dietary supplementation at physiological concentrations found in plasma. They reported that only EQ (10 µM) down-regulates NO and PGE_2_ levels in RAW 264.7 cells that are LPS-stimulated [41].

Docking studies show that R-EQ is related to iNOS–ligand complexes. It reaches Phe369 from human iNOS, corresponding to Phe363 murine iNOS, which belongs to the enzyme’s active site.

It is well known that NOS isoenzymes have a structurally similar active site due to their conserved protein sequence, in which the N-terminal catalytic oxygenase module binds to the HEME group (protoporphyrin IX-containing Fe^+2^) [53]. Also, it has been established that the iNOS active site shows oxygenase and reductase domains, which are catalytically active sites when separated. The second step of NO synthesis is reconstitution, which combines the reductase and oxygenase domains of human eNOS and murine iNOS.

On the other hand, it is well known that the inflammatory response is different in each disease. However, it can be characterized by the presence of TNF-α and IL-6. In this study, treatment with DZ and S-EQ down-regulated TNF-α in agreement with the reported by Kang et al., who demonstrated that EQ inhibited both TNF-α production and mRNA expression in RAW264.7 cells in a concentration-dependent way [54]. Similarly, EQ and DZ decreased IL-6 levels, a cytokine often used as a marker for systemic activating pro-inflammatory cytokines [55]. Both cases could be caused by transcription factors that interfere with protein expression.

Emerging studies have also shown that LPS-induced inflammation activates the NLRP3 inflammasome in macrophages [56], which is essential in provoking inflammatory responses. It serves as a molecular platform that mediates the auto-activation of caspase 1, which cleaves the pro-forms of IL-1β and IL-18 to active forms because NLRP3 recruits ASC and procaspase 1 to form an inflammasome complex [3,6,57].

Among the members of NLR, NLRP3 is the most characterized one due to its association with several immune, inflammatory, and auto-inflammatory diseases [57,58] and a wide array of microbial and non-microbial origin activators. Also, the inflammation induced by LPS in macrophages has been related to NLRP3 activation.

This study shows that LPS up-regulates the NLRP3 protein expression in murine peritoneal macrophages, an effect inhibited by DZ and S-EQ treatments. These results agree with those from Zhou et al. [59], who demonstrated that a DZ compound called X-11-5-27 restrains the formation of the NLRP3 inflammasome and maturation of IL-1β.

The NLRP3 inflammasome has been associated with the pathogenesis and development of autoimmune diseases, including rheumatoid arthritis (RA), systemic lupus erythematosus (SLE), and ankylosing spondylitis (AS), among others. In RA, this inflammasome is up-regulated in monocytes, macrophages, and dendritic cells; it also increases caspase-1 and IL-18 and negatively regulates the NF-κB pathway.

In the case of SLE, multiple mechanisms are involved, including an up-regulated NLRP3, caspase-1, and IL-1β in monocytes, enhancing Th17 cell polarization and activating necroptosis, events that improve disease activity. Similar mechanisms are related in AS, in which NLRP3, ASC, caspase-1, IL-1β, IL-17A, and IL-23 are up-regulated in the peripheral blood mononuclear cells (PBMCs) of AS patients, indicating that activation of the NLRP3 may trigger AS and enhance the responses of Th17 cells [4].

In addition, our work group previously demonstrated in a chronic ulcerative colitis model that the NLRP3 inflammasome (canonical and non-canonical pathways) is inhibited by apigenin administration by down-regulating IL-1β and IL-18 due to regulation of cleaved caspase 1 [60].

Caspase 1 is an inactive zymogen in the cytosol of phagocytic cells. During stimulation, the dormant pro-caspase 1 zymogen is self-activated by proteolytic cleavage into the enzymatically active heterodimer (10 and 20-kDa subunits). Also, when the inflammasomes recruit the pro-enzyme through its CARD domain, it gains proteolytic activity, inducing “pyroptosis,” an inflammatory type of cell death [61,62].

Our results demonstrated that DZ and S-EQ did not affect caspase 1 protein expression. However, both compounds decrease IL-1β and IL-18 levels. This effect agrees with previous reports about caffeic acid phenethyl ester, demonstrating that this compound does not inhibit caspase 1 enzyme activity but preferentially targets ASC and NLRP3 [63].

Similarly, Zhou et al. [59] reported that NLRP3 is considered an essential pattern-recognition receptor that participates in developing acute gouty arthritis. In addition, the docking studies demonstrated that 4-(2-(4-chlorophenyl)-1-((4-chlorophenyl)amino)ethyl)benzene-1, 3-diol (CBED) targets inside the ATP-binding pocket of NLRP3, forming hydrogen bonds between Glu539, Asp750, and His724. In this sense, DZ and EQ showed the highest in silico affinity to NLRP3 protein compared to ASC or caspase 1. Therefore, according to molecular docking predictions, DZ and EQ may modulate the abovementioned protein activity as we have hypothesized.

A molecular docking approach was utilized to support our second hypothesis. DZ and EQ were coupled to a well-known transcriptional factor NF-κB. Both experimental and molecular docking results suggest that DZ and EQ could be exerting moderate inhibitory activity over NF-κB, affecting the transcription of the genes involved in inflammation, such as COX-2, iNOS, TNF-α, IL-6, IL-1β, and IL-18, as well as the indirect transcriptional regulation over inflammasome components including NLRP3 [17,32]. Both DZ and EQ reach the interface of the P65 and P50 NF-κB dimer and are stabilized by hydrogen bonds, mainly with Cys197 in P65 and Lys278 in P50, indicating that both interactions are essential for recognition.

Although several reports have demonstrated that the docking procedure reproduces the experimental ligand–protein affinity [64], ex vivo experiments are necessary to corroborate our postulate.

Additionally, considering the in vivo importance of drugs’ physicochemical properties, lipophilicity, defined as log *p*, is the most important; it can also describe pharmacodynamics, pharmacokinetics, and toxic aspects of the drug activity [65]. As shown previously, the DZ biotransformation to metabolite EQ increases lipophilicity, possibly improving the pharmacokinetic absorption during in vivo administration.

This biotransformation is essential since it has been reported that the presence of an “equol-producer” or “non-equol producer” is influenced by dietary and food matrix factors, among other things [23,66].

It is essential to highlight that our results contribute to the knowledge of the anti-inflammatory and autoimmune mechanisms of action of two well-known isoflavones, daidzein and equol, emphasizing their usefulness as natural alternatives for chronic inflammatory diseases. However, deepening the investigations for human purposes is necessary, considering all factors involved in the pharmacokinetic process and its biological factors, mainly biotransformation ones.

## 4. Materials and Methods

### 4.1. Chemicals

DZ (purity 98%) and S-EQ (purity ≥ 97%) were obtained from Alfa Aesar (Tewksbury, MA, USA) and Sigma-Aldrich (Merck KGaA, Darmstadt, Germany). Stock solutions were prepared freshly in dimethyl sulfoxide (DMSO) (Panreac, Barcelona, Spain). From this solution, the corresponding experimental dilutions were prepared in a 5% FCS RPMI 1640-supplemented medium, considering a DMSO concentration of ≤ 1%, which does not influence cell response.

### 4.2. Ex Vivo Evaluation

#### 4.2.1. Animals

Six eight–ten-week-old female Swiss mice (20–30 g) were provided by Harlan Interfauna Ibérica (Barcelona, Spain). They were housed randomly, with 3 mice/cage, at a constant temperature between 20 and 25 °C, 40–60% humidity, and a 12 h light/dark cycle. During all experiments, animals had ad libitum access to water and feed (standard rodent chow) (Panlab A04, Seville, Spain). Animals were acclimated for one week before the experiments.

All animal care and experimental procedures complied with the European Union’s guidelines regarding animal experimentation (Directive of the European Counsel 2012/707/EU) and a protocol approved by the Animal Ethics Committee of the University of Seville (CEEA-US2018-11/2) and with the approval of the Junta de Andalucia 23/07/2018/119.

#### 4.2.2. Obtention of Murine Peritoneal Macrophages Cultures

Animals were intraperitoneally administered with 1 mL of sterile thioglycolate medium (10% *w*/*v*) (Scharlau, Barcelona, Spain) to encourage the presence of macrophages in the peritoneum. Daily changes in behavior, food, and water consumption; body weight; and survival were monitored until sacrifice. Three days after injection, mice were euthanized by CO_2_ exposure. Washing the peritoneal cavity with cold PBS was achieved to obtain peritoneal exudate cells [41]. Samples were centrifugated, and the cells were resuspended in 10% heat-inactivated fetal calf serum (FCS) RPMI 1640-supplemented medium (PAA^®^, Pasching, Austria), 2 mM l-glutamine, 4.5 g/L of glucose, 10 mM HEPES buffer, streptomycin (100 mg/mL), and penicillin (100 U/mL) (PAA^®^ Pasching, Austria).

1 × 10^6^ cells/mL were incubated for 2 h at 37 °C in a 5% CO_2_ humidified atmosphere. After incubation, non-adherent cells were discarded, and remanent cells were washed with cold PBS. Treatments with DZ (25, 50, and 100 µM) or EQ (5, 10, and 25 µM) were added diluted in a 5% FCS RPMI 1640-supplemented medium. After 30 min, murine peritoneal macrophages (1 x 10^6^ cells/mL) were treated with 5 µg/mL of LPS from *E. coli* (Sigma-Aldrich^®^, St Louis, MO, USA) for 18 h. In each experiment, viability was always ≥95%. These concentrations were used considering previous in vitro reports, in which DZ is evaluated in the range of 25–160 µM [67,68] and EQ in the approximate range of 1.25–41.6 µM (0.3, 1, 3, and 10 µg/mL) [69].

#### 4.2.3. Cell Viability

In total, 1 x 10^6^ cells were incubated (18 h) with DZ and EQ treatments at 25, 50, and 100 µM and 5, 10, and 25 µM, respectively, and cell growth/viability was assessed by sulforhodamine B (SRB) assay [70]. The fixation of adherent cells was achieved by adding 50% (*w*/*v*) of cold trichloroacetic acid (Sigma-Aldrich, St Louis, MO, USA) (50 μL) and a period of incubation (60 min, 4 °C). After discarding the supernatant, 5 washes with deionized water were realized. A volume of 100 μL of 0.4% (*w*/*v*) of SRB in 1% acetic acid (Panreac, Barcelona, Spain) was added per well, and then plates were incubated (30 min, room temperature).

Five washes with a 1% acetic acid solution were realized, and finally, 100 μL/well of 10 mmol/l Tris base, pH 10.5 (Sigma-Aldrich, St Louis, MO, USA) was added. An ELISA reader at 492 nm (BioTek, Bad Friedrichshall, Germany) was used to obtain sample absorbances. Cell growth/viability was reported considering the absorbance of non-treated cells as 100%.

#### 4.2.4. Measurement of Nitrite Production

According to Section 4.2.2, cells were exposed to DZ (25, 50, and 100 µM) or EQ (5, 10, and 25 µM) for 30 min and then LPS-stimulated during 18 h. Griess reaction [71] was achieved by a mixture of 100 µL of supernatants and the same volume of Griess reagent (Sigma^®^, St Louis, MO, USA) freshly prepared. After 15 min of incubation (darkness and room temperature), the absorbance was measured at 540 nm with an ELISA reader (BioTek, Bad Friedrichshall, Germany). As an index of NO generation, the amount of nitrite was determined and expressed as the nitrite production percentage compared with that of stimulated non-treated cells.

#### 4.2.5. Inflammation Mediators’ Quantification

Levels of cytokines IL-1β and tumor necrosis factor (TNF)-α (R&D Systems^®^, Inc., Minneapolis, MN, USA) and IL-6 (Diaclone) were obtained according to methods indicated in the corresponding ELISA kits. PGE_2_ was quantified by an EIA kit (Cayman Chemical Company, Ann Arbor, MI, USA).

Moreover, we used supernatants collected from macrophages treated with DZ 100 µM and EQ 25 µM as samples.

#### 4.2.6. Isolation of Cytoplasmic Proteins and Immunoblotting Detection

After macrophage stimulation and incubation (Section 4.2.2), cells were obtained by rinsing, scraping, and resuspending in an ice-cold PBS solution (added with protease and phosphatase inhibitors) to isolate cytoplasmatic proteins [72]. According to Bradford’s method, we established samples’ total protein content using a protein assay reagent (BioRad, Hercules, CA, USA) and a standard of γ-globulin [73].

Sample aliquots containing a standard 20 µg of protein content were separated by sodium dodecyl sulfate-polyacrylamide gel electrophoresis in a 10% acrylamide gel. Then, they were transferred by electrophoresis onto nitrocellulose membranes, which were incubated overnight (4 °C) in the presence of primary antibodies (Table 2).

Subsequently, membranes were rinsed and incubated with secondary antibodies that were horseradish peroxidase (HRP)-labeled (Table 2) in a blocking solution during 1–2 h (room temperature).

An anti-β-actin antibody (Sigma Aldrich, St. Louis, MO, USA) was used to analyze β-actin expression to show equal protein loading in the blots. Then, a chemiluminescence light-detecting kit (Pierce, Rockford, IL, USA) was used, and immunosignals were obtained using the indications from Fujifilm Image Reader (Stamford, CT, USA) in an LAS-3000 Imaging System. After normalization with the housekeeping loading control, densitometry data were analyzed. Results were expressed and compared with those of stimulated cells using the Image J program (Public Domain, imagej.net).

#### 4.2.7. Statistical Evaluation

Data obtained were reported as means ± standard error (SEM) for at least three independent experiments. *p* < 0.05 statistical significances were obtained with the Graph Pad Prism^®^ Version 5 software (San Diego, CA, USA) by a one-way ANOVA (analysis of variance) and a post hoc analysis by the Student–Newman–Keuls test.

### 4.3. In Silico Evaluation

#### 4.3.1. Protein Models

Sequences of primary proteins were retrieved from the UniProt database (https://www.uniprot.org/ (accessed on 5 January 2023)). Furthermore, a sequence alignment using BLAST protein [74] was performed to find the available protein structures (3D) on the protein data bank (PDB) [75]; the 3D structures with the best resolution and the most complete sequence were taken for study (see Table 3). The proteins retrieved from PDB were stripped from molecules out of interest, like water, acetate, phosphate, or any other crystallization artifacts. Procaspase 1 was further refined since two loop regions were missing, spanning Gln103 to Thr125 and Val299 to Ser307; the loop structure refinement was performed using Modeller v 9.19 [76]. Through homology modeling, NLRP3 protein was modeled using Modeller v 9.19 from Asp135 to Trp1036 as a template. The crystal structure of rabbit NOD2 was used (PDB: 5IRM), which shared a homology of 30% (data not published yet). NLRP3 and procaspase 1 were further submitted to successive refinements in the ModRefiner online server (https://zhanglab.ccmb.med.umich.edu/ModRefiner (accessed on 5 January 2023)) until optimal model quality was obtained. The model quality was evaluated on the MolProbity online server.

Proteins were further prepared on AutoDockTools-1.5.6 [35], where only polar hydrogens were considered, and Kollman charges [29] were assigned to generate a PDBQT format for docking studies.

#### 4.3.2. Docking Analysis

DZ and S-EQ were drawn using Gauss View 5.0 (Gaussian, Inc., Wallingford, CT, USA) [77], and further geometrical optimization was carried out using Gaussian 09 (Gaussian, Inc., Wallingford, CT, USA) [78], under vacuum, and the semi-empirical method AM1 was used. The output file was converted into a PDB file using Gauss View 5.0 [77]. It was processed in AutoDock Tools 1.5.6 (Molecular Graphics Laboratory, La Jolla, CA, USA) [35]. Only hydrogen atoms (polar) and flexible bonds were considered during the calculation. Partial Gasteiger atomic charges were assigned to the compound. Blind docking was carried out for the NLRP3 protein and Autodock Vina software 1.1.2 (Molecular Graphics Laboratory, La Jolla, CA, USA) [79] was used because this software allows for the exploration of more extensive systems with a more excellent grid box than Autodock 4.2 (Molecular Graphics Laboratory, La Jolla, CA, USA) [35]. For proteins ASC, pro-caspase 1, caspase 1, COX-2, and iNOS, AutoDock 4.2 (Molecular Graphics Laboratory, La Jolla, CA, USA) [35] was employed, with a grid box of 126 Å3, and in all the cases the grid box was placed in the center of the protein with a grid spacing of 0.375 Å3 [46,80].

We established as parameters an initial population of 100 individuals placed randomly, 27,000 generations, 100 generations for picking the worst individual, and a maximum number of energy evaluations (1 × 10^7^) for the Lamarckian Genetic Algorithm method. Default values were maintained for all other parameters.

For analysis, we obtained the lowest free energy for each complex, which was visualized after with the Autodock Tools 1.5.6 [34] Chimera V1.11.2 (UCSF Resource for Biocomputing, Visualization, and Informatics, National Institutes of Health, University of California, San Francisco, CA, USA) [81] and Discover Studio v17.2 (Dassault Systèmes BIOVIA, 2016). Two-dimension (2D) maps of interaction were retrieved from Discover Studio v17.2 (Dassault Systèmes BIOVIA, 2016).

#### 4.3.3. Log *p* Value Estimation and Electrostatic Potential Map Characterization

The estimation of the decimal logarithm of the partition coefficient (log *p*) was performed using the online VCCLAB program (German Research Center for Environmental Health (GmbH), Neuherberg, Germany) [82,83]. On the other hand, the electrostatic potential map was obtained with ArgusLab 4.0.1 Software, in which we selected 0.002 au (atomic units) as the isodensity surface. The images represented in white color represent the most positive electronegativity potential, while the red color indicates the most negative one. Values were obtained using an electron volt (eV) scale related to a color scale.

## 5. Conclusions

This study establishes that DZ and its active EQ metabolite inhibit LPS-induced inflammatory responses in peritoneal murine macrophages via down-regulation of NO and PGE_2_ generation. These protective effects seem to result from decreasing iNOS and COX-2 protein expression, respectively. Furthermore, we demonstrated that DZ and EQ anti-inflammatory activity is related to inhibiting the canonical inflammasome pathway, regulating NLRP3 and consequently decreasing IL-1β and IL-18 activation. Also, considering the concentrations used, EQ was more potent than DZ, corroborating the importance of the biotransformation process and the chemical affinity for the NLRP3 protein and other inflammatory components, such as COX-2 and iNOS enzymes. These results contribute to understanding DZ and EQ’s possible mechanisms of action and information that could support their use as inflammasome inhibitors and their potential application in chronic inflammatory and autoimmune diseases.

## Figures and Tables

**Table 1:** DZ and EQ residue interactions.

	Daidzein			S-Equol		
	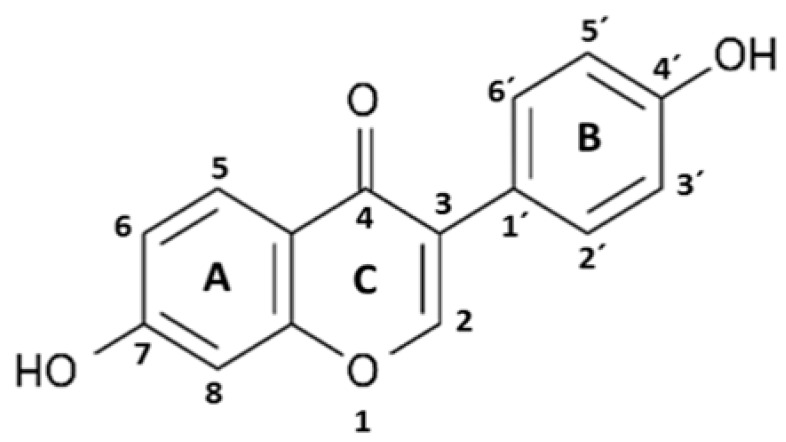			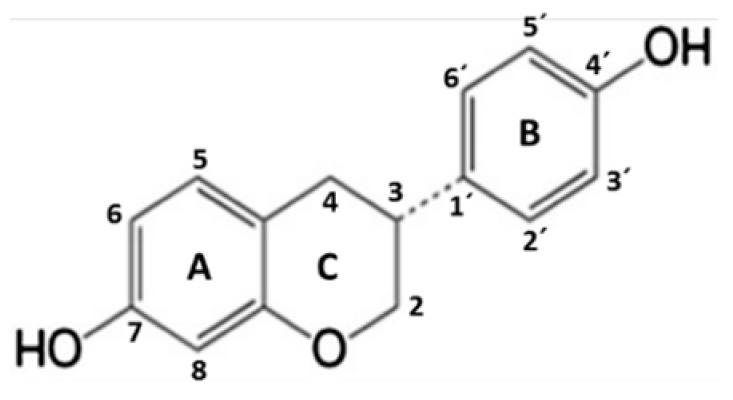		
Protein	ΔG *	Ki **	Residue Interactions	ΔG *	Ki **	Residue Interactions
*COX-2*	−8.61	0.49	Ile124, Asp125, Thr129, Thr149, Arg150, Ala151, Asn375, Arg376, Ala378, Arg469, Phe529	−8.57	0.52	Ile124, Ser126, Pro128, Asn375, Arg376, Ile377, Ala378, Phe381, Lys532, Gly533, Phe529
*iNOS*	−7.56	2.85	Gln263, Tyr347, Pro350, Ala351, Val352, Phe369, Asn370, Tyr373, Glu377, Asp382, Arg388, Hem510	−8.60	0.50	Pro350, Ala351, Val352, Gly371, Trp372, Tyr373, Glu377, Asp382, Hem510
*NLRP3*	−7.80	1.91	Ser374, Glu375, Arg675, Gly719, Leu720, Ser752, Leu753, Gly754, Asp795, Pro796, Gly915, Leu916, Ser917, Leu810, Gly811, Phe1113	−7.50	3.17	Leu187, Leu188, Ala189, Ile190, Glu197, Pro199, Val200, Ser201, Ile203, Pro211, Asp212, Leu822
*ASC*	−5.57	83.41	Tyr36, Gly37, Ile39, Pro40, Phe59, Tyr60	−5.95	43.81	Lys109, Pro110, Leu112, His113, Phe114, Ile115, Asp116, Arg119, Arg160, Phe163, Ser164
*Pro-caspase 1*	−7.23	5.03	Lys268, Asn269, Gly303, Thr309, Thr310, Glu312, Phe313, Glu314, Lys320	−6.12	32.45	Lys268, Thr309, Thr310, Glu311, Glu312, Glu314, Lys319
*Caspase 1*	−7.60	2.70	Arg240, Cys285, Arg286, Ala284, Cys331, Glu241, Gly242, Gln257, Leu258, Ile282	−6.42	19.80	Arg179.Ser236, His237, Gln283, Cys285, Ser339, Trp340, Arg341
*NF-κB*	−5.43	104.92	P65: Cys197, Arg198 P50: Lys278, Thr304, Val306, His307, Arg308	−5.44	102.86	P65: Cys197, Arg198 P50: Lys278, Thr304, Val306, His307, Arg308

* Free energy (Kcal/mol); ** inhibition constant (μM).

**Table 2:** Characteristics of specific primary antibodies used.

Antibody	Type	Supplier	Dilution
Rabbit polyclonal anti-COX-2	Primary	Cayman^®^, Ann Arbor, MI, USA	1:2500
Rabbit polyclonal anti-iNOS	Primary	Cayman^®^, Ann Arbor, MI, USA	1:1000
Rabbit polyclonal anti-IL-18	Primary	Abcam plc	1:200
Rabbit anti-ASC	Primary	Santa Cruz Biotechnology^®^, Inc.	1:100
Rabbit anti-caspase 1	Primary	Novus Biologicals, LLC	1:400
Mouse anti-NLRP3	Primary	Novus Biologicals, LLC	1:100
Anti-rabbit	Secondary	Cayman^®^, Ann Arbor, MI, USA	1:2500
Anti-mouse	Secondary	Dako^®^, Atlanta, GA, USA	1:2500

**Table 3:** Three-dimensional (3D) structures of the protein used in the present study.

Protein	UniProt	PDB (Resolution in Å/Quality Model)
NLRP3	Q96P20	NA ^a^ (98%)
ASC	Q9ULZ3	2KN6
Pro-caspase 1	P29466	3E4C (2.05/98%)
Caspase 1	P29466	1RWK (2.30)
COX-2	Q05769	5COX (3.00)
NF-κB	Q04206 and P19838	1NFI (2.70)

^a^ NA: not available.

## Data Availability

The data presented in this study are available on request from the corresponding author due to privacy restrictions.
